# Distinction and mutual Influences between Parkinson's Disease‐related and unrelated Chronic Pain

**DOI:** 10.1002/mdc3.70262

**Published:** 2025-08-20

**Authors:** Jonathan Hunger, Florian Brugger, Georg Kägi, Jens Carsten Möller, Nathalie Hollenstein, David H. Benninger, Michele Tinazzi, Julien F. Bally, Roman Gonzenbach, Daniel Ciampi de Andrade, Santiago Perez Lloret, Veit Mylius

**Affiliations:** ^1^ Department of Neurology Center for Neurorehabilitation Valens Switzerland; ^2^ Department of Neurology Kantonsspital St. Gallen Switzerland; ^3^ Department of Neurology Inselspital, Bern University Hospital, and University of Bern Bern Switzerland; ^4^ Department of Neurology Philipps University Marburg Germany; ^5^ Parkinson Center, Center for Neurological Rehabilitation Zihlschlacht Switzerland; ^6^ Service of Neurology and Stroke Unit HFR Fribourg–Cantonal Hospital Fribourg Switzerland; ^7^ Service de Neuroréadaptation HFR Meyriez‐Murten Meyriez Switzerland; ^8^ Service of Neurology, Department of Clinical Neurosciences Lausanne University Hospital and University of Lausanne Lausanne Switzerland; ^9^ Center for Parkinson and Movement Disorders Reha Rheinfelden Switzerland; ^10^ Department of Neurology University of Basel Basel Switzerland; ^11^ Department of Neurosciences, Biomedicine and Movement University of Verona Verona Italy; ^12^ Department of Health Science and Technology, Faculty of Medicine, Center for Neuroplasticity and Pain (CNAP), Aalborg University Aalborg Denmark; ^13^ Instituto Universitario de Ciencias de la Salud, Fundación H.A Barceló, Consejo de Investigaciones Científicas y Técnicas (CONICET) Buenos Aires Argentina; ^14^ Departamento de Fisiología, Facultad de Medicina Universidad de Buenos Aires Buenos Aires Argentina; ^15^ Department of Neurology Kantonsspital Graubünden Chur Switzerland

**Keywords:** chronic pain, Parkinson's disease, PD‐related pain, PD‐unrelated pain, nonmotor symptom

## Abstract

**Background:**

Chronic pain (i.e. > 3 months) is a common nonmotor symptom in patients with Parkinson's disease (PD), but the attribution to PD is critical for further treatment.

**Objectives:**

We explored the PD Pain Classification System (PD‐PCS) criteria for the diagnosis of PD‐related pain and mutual influences between PD‐related and PD‐unrelated pain.

**Methods:**

In this multicenter study, 120 nondemented PD patients were assessed using the PD‐PCS as well as motor and nonmotor questionnaires. The PD‐PCS consists of 3 steps: first, it classifies chronic pain as unrelated or related to PD according to 1 of 4 criteria (at onset or aggravated by PD, in the *off* phase, improvement with dopaminergic treatment, and with dyskinesia); second, it allows the classification of pain mechanisms (neuropathic, nociceptive, and nociplastic); and finally, it provides a score.

**Results:**

Chronic pain was present in 92% of patients, with PD‐related pain in 73% and non‐PD‐related pain in 53%. Higher PD‐PCS scores were reported when PD‐related pain was present. In cases of concurrent PD‐related and PD‐unrelated pain (35%), there was a moderate correlation between pain severity. Improvement with dopaminergic medication and pain in the *off* phases were the most common factors defining an association of pain with PD. These factors often occur together, whereas pain during dyskinesia occurs independently.

**Conclusion:**

The PD‐PCS criteria allow differentiation between PD‐related and PD‐unrelated chronic pain through 2 approaches, assessing periods of either low or high dopaminergic stimulation. PD‐unrelated pain should also be taken into account, as it is more common than in the general population and as it may influence PD‐related pain.

Chronic pain, referring to pain lasting >3 months and being present most of the days,^1^ represents one of the most prevalent nonmotor symptoms (NMS) in patients with Parkinson's disease (PD), affecting quality of life.^2^ Previous estimations for the prevalence of PD‐related chronic pain range from 60% to 90%,^3^, ^4^, ^5^, ^6^, ^7^ whereas a range from 20% to 60% has been given for PD‐unrelated pain.^5^, ^6^ However, since 18% (10–29) of the elderly population also suffers from chronic pain,^8^ chronic pain related to or aggravated by PD is often not adequately diagnosed and treated.^9^, ^10^ For example, nonsteroidal anti‐inflammatory drugs with potential side effects are often prescribed in clinical practice, as pain is not always considered an NMS in PD.^9^ In addition, reciprocal influences emerged in shoulder and back pain as well as in posture abnormalities and musculoskeletal (MSK) pain.^11^, ^12^, ^13^

However, so far, it has been difficult to differentiate both types of pain. Nonmotor questionnaires attribute pain to PD either by the exclusion of other causes^14^ or by defining them as pain during the *off* stage and improved by dopaminergic treatment.^15^ The most recent NMS scales include drug response fluctuations by assessing either changes between *on* and *off* ^16^, ^17^ or both conditions separately.^18^ Based on this approach, the King's PD Pain Questionnaire was the first validated questionnaire to include any unexplained pain in the past month and to categorize pain into 7 domains (ie, MSK, chronic, fluctuation‐related, nocturnal, orofacial, discoloration or edema/swelling, and radicular pain).^19^

The PD Pain Classification System (PD‐PCS) was later developed and validated to specifically assess chronic pain in people with PD and to further differentiate PD‐related (ie, pain at the beginning or aggravated by PD, pain aggravated during the *off* phase, pain during dyskinesia during the *on* phase, or pain improved by dopaminergic medication) from PD‐unrelated pain before hierarchically attributing the pain syndrome to 1 of the 3 pain mechanisms (ie, nociceptive, neuropathic, and nociplastic)^6^, ^20^ and providing a score.

The PD‐PCS original study was a multicenter study and included 70 patients from Swiss neurology clinics. In this validation study, the focus was on people with PD‐related pain, whereas those with PD‐unrelated pain and those with comorbid chronic pains (both PD‐related and PD‐unrelated occurring in the same patient) were not further characterized. For the present study, we extended the sample to 120 patients and described the characteristics and diagnostic criteria for chronic PD‐unrelated pain, compared it to chronic PD‐related pain, and explored their mutual influences when both co‐occurred in the same individuals.

## Patients and Methods

### Participants

Consecutive inpatients and outpatients with or without pain and with the clinical diagnosis of PD according to the criteria of the United Kingdom PD Society Brain Bank^21^ were recruited in 4 centers in Switzerland (Center for Neurological Rehabilitation in Zihlschlacht, Department of Neurology of Kantonsspital St. Gallen, Service of Neurology of Lausanne University Hospital, and Center for Neurorehabilitation in Valens). Participants were screened for dementia using the Mini‐Mental Status Examination (exclusion criterion, cutoff <25) before participation. Patients receiving deep brain stimulation (n = 12) but not levodopa‐carbidopa intestinal gel (LCIG) and apomorphine were included in the study. Informed consent was obtained before participation.

### Clinical Assessments

This was a cross‐sectional, national, multicenter study. Part of the data (N = 70) has been evaluated as part of the international validation study of the PD‐PCS.^6^ PD patients were assessed in the *on* state by neurologists specialized in movement disorders and trained in the use of the PD‐PCS Questionnaire. Raters assessed patients' pain in a standardized way using the PD‐PCS. The PD‐PCS was assessed separately for each pain syndrome, and up to 4 pain syndromes (associated or not associated with PD) were documented and analyzed. In addition to the previous publication, which reported only 1 pain syndrome in each category of PD‐related pains and up to 2 nonrelated pains,^6^ we assessed up to 3 different pains in each category (not exceeding 4 pains).

General information on PD history was obtained. Medication intake was recorded (levodopa equivalents were calculated according to Tomlinson et al.^22^). Then, patients completed the following questionnaires and tests together with the examiner: PD‐PCS,^6^ QoL in the Parkinson's Disease Questionnaire 8‐Item version (PDQ‐8),^23^ McGill Pain Questionnaire short‐form 1,^24^ Hospital Anxiety and Depression Scale (HADS),^25^ and the Wearing‐Off Questionnaire 9‐Item version (WOQ‐9).^26^ Finally, the Movement Disorders Society revision of the unified Parkinson's Disease Rating Scale, Parts III and IV (MDS‐UPDRS III and IV) were evaluated.^27^

### The PD‐PCS Questionnaire

The PD‐PCS questionnaire is a rater‐based scale developed and validated by an international group.^6^, ^20^ The first part addresses demographics and disease characteristics, followed by the localization and duration of up to 4 pains.

The association of the respective pain syndrome with PD is given if 1 of 4 criteria applies. These include (1) pain beginning together with first motor symptoms or an increase in previous pain by PD, (2) pain when symptoms of PD aggravate (ie, during the *off* phase), (3) pain during dyskinesia (ie, during the *on* phase or biphasic), or (4) pain that improves considerably with dopaminergic medication. A comprehensive description of each criterion is provided in the validation study.^6^

Thereafter, the respective chronic pain is classified according to its mechanistic descriptor (neuropathic, nociceptive, or nociplastic) using a hierarchical approach: in the first step, we define neuropathic pain using a screening questionnaire (*questionnaire douleur neuropathique 4 (DN4)*).^28^ According to its distribution, neuropathic pain can be further subdivided into central and peripheral. However, central neuropathic pain is considered a rare entity, and it can be assumed that these patients rather fulfill the criteria of nociplastic pain.^29^ This approach follows the grading approach to the diagnosis of neuropathic pain and classifies pain as probable neuropathic pain.^30^ When the pain syndrome does not fulfill these criteria, the patient will be screened for nociceptive pain if the pain is perceived in muscles, tendons, or fascia or if the patient has painful rigidity or palpation thereof is painful. These comprise MSK pains due to motor fluctuations such as *on* period pain (eg, early‐morning pain, wearing‐off or end‐of‐dose pain) or sudden‐off pain, many painful dystonic spasms (early‐morning dystonia, off‐period dystonia, beginning‐of‐dose dystonia, end‐of‐dose dystonia), and peak‐of‐dose or biphasic pains (mostly associated with dyskinesia). Localized or regional pain syndromes, the myofascial pain syndrome, and coat‐hanger headaches (neck pain in hypotension) are included in this category.^31^, ^32^ If these 2 categories are excluded, and further neuropsychiatric symptoms occur concurrently, the pain syndrome can be classified as nociplastic.^33^, ^34^ We included the nonmotor‐off, leg motor restlessness, the dopaminergic dysregulation syndrome, and the dopamine agonist withdrawal syndrome in this category.^6^

For each pain syndrome, a severity score based on pain intensity (0–10) multiplied by its frequency and impact in daily living (each using a 3‐point Likert score) can be obtained, so scores can range from 0 to 90.

If pain is not associated with PD, causes, mechanisms, and pain ratings were also assessed using the PD‐PCS questionnaire.

### Sample Size

We calculated that 120 patients would be enough to find statistically significant differences of at least 0.5 standardized units in numerical variables and of at least 25% in categorical variables, with 80% power and 5% α error.^35^

### Statistical Analysis

The centers’ clinical data were deidentified and transferred to the Instituto Universitario de Ciencias de la Salud, Fundación H.A Barceló (Buenos Aires, Argentina) for statistical analysis. Data are shown as mean ± standard deviation, except otherwise indicated. Between‐group comparisons of numerical variables were performed using Student's *t*‐test or analysis of variance (ANOVA) and χ^2^ test for categorical ones. If the tests' assumptions were not met, nonparametric tests were used. For ANOVA, post hoc testing was performed using Tukey's honestly significant difference (HSD) test. Correlation coefficients were calculated using Pearson's parametric method. Statistical analysis was performed with R 4.4.1 (The R Foundation, Vienna, Austria) using the following libraries: tidyverse, table1, rstatix, modelbased, and ggpattern.

## Results

### Participant Characteristics

A total of 120 PD patients were recruited from 4 centers in Switzerland. Mean age was 69.9 ± 8.6, and 74 (61.7%) patients were men. Mean disease duration and pain duration were 7.3 ± 4.9 and 7.7 ± 10.5 years, respectively. The mean MDS‐UPDRS III score in the *on* state was 30.1 ± 13.7. There was a weak correlation between the MDS‐UPDRS III score and age (r = 0.22, *P* = 0.02) but no correlation with disease duration (r = −0.01, *P* = 0.27) or PD‐PCS total score (r = −0.02, *P* = 0.87). According to the WOQ‐9, 107 (92%) patients experienced either motor or nonmotor fluctuations; 32 (27.1%) patients had choreatic dyskinesia, and 41 (35.7%) experienced *off* periods, as assessed using the MDS‐UPDRS IV. More information on the sample is provided in Table 1.

**TABLE 1:** Characteristics of the sample

Variable	PD sample (N = 120)
Age	69.9 ± 8.64
Sex	
Male	74 (61.7%)
Female	46 (38.3%)
Years since PD diagnosis	7.25 ± 4.90
Medication	
Levodopa	117 (97.5%)
Dopamine agonists	68 (56.7%)
MAO‐B inhibitors	21 (17.5%)
COMT inhibitors	32 (26.7%)
Amantadine	8 (6.7%)
LDED	840 ± 414
Analgesics	31 (25.8%)
Antidepressants	5 (4.2%)
MDS‐UPDRS III	30.1 ± 13.7
MDS‐UPDRS IV	4.29 ± 4.07
MDS‐UPDRS IV duration of dyskinesia	
No dyskinesias	86 (72.9%)
1%–25%	25 (21.2%)
26%–50%	3 (2.5%)
51%–75%	4 (3.4%)
MDS‐UPDRS IV *off* time during the waking day	
No *off* time	74 (64.3%)
1%–25%	23 (20.0%)
26%–50%	7 (6.1%)
51%–75%	6 (5.2%)
76%–100%	5 (4.3%)
Wearing‐Off Questionaire	4.28 ± 2.34
Presence of motor or nonmotor fluctuations	107 (91.5%)
MMSE	28.3 ± 1.72
PDQ‐8	8.83 ± 5.20
Hospital Anxiety Scale	5.73 ± 3.96
Above cutoff	31 (25.8%)
Hospital Depression Scale	5.54 ± 3.39
Above cutoff	30 (25.0%)
Wearing‐Off Questionnaire 9‐Pain/aching	
No	32 (26.9%)
Yes, and usually improves after PD medications	54 (45.4%)
Yes, and does not improve after PD medications	33 (27.7%)

Abbreviations: PD, Parkinson's disease; MAO‐B, monoamine oxidase‐B; COMT, catechol‐*o*‐methyltransferase; LDED, levodopa equivalent dose; MDS‐UPDRS, Movement Disorder Society‐unified Parkinson's Disease Rating Scale; MMSE, Mini‐Mental State Examination; PDQ‐8, Parkinson's Disease Questionnaire 8‐Item version.

### Characteristics of Participants with Chronic PD‐Related Pain, PD‐Unrelated Pain, or Both

**FIG. 1:**
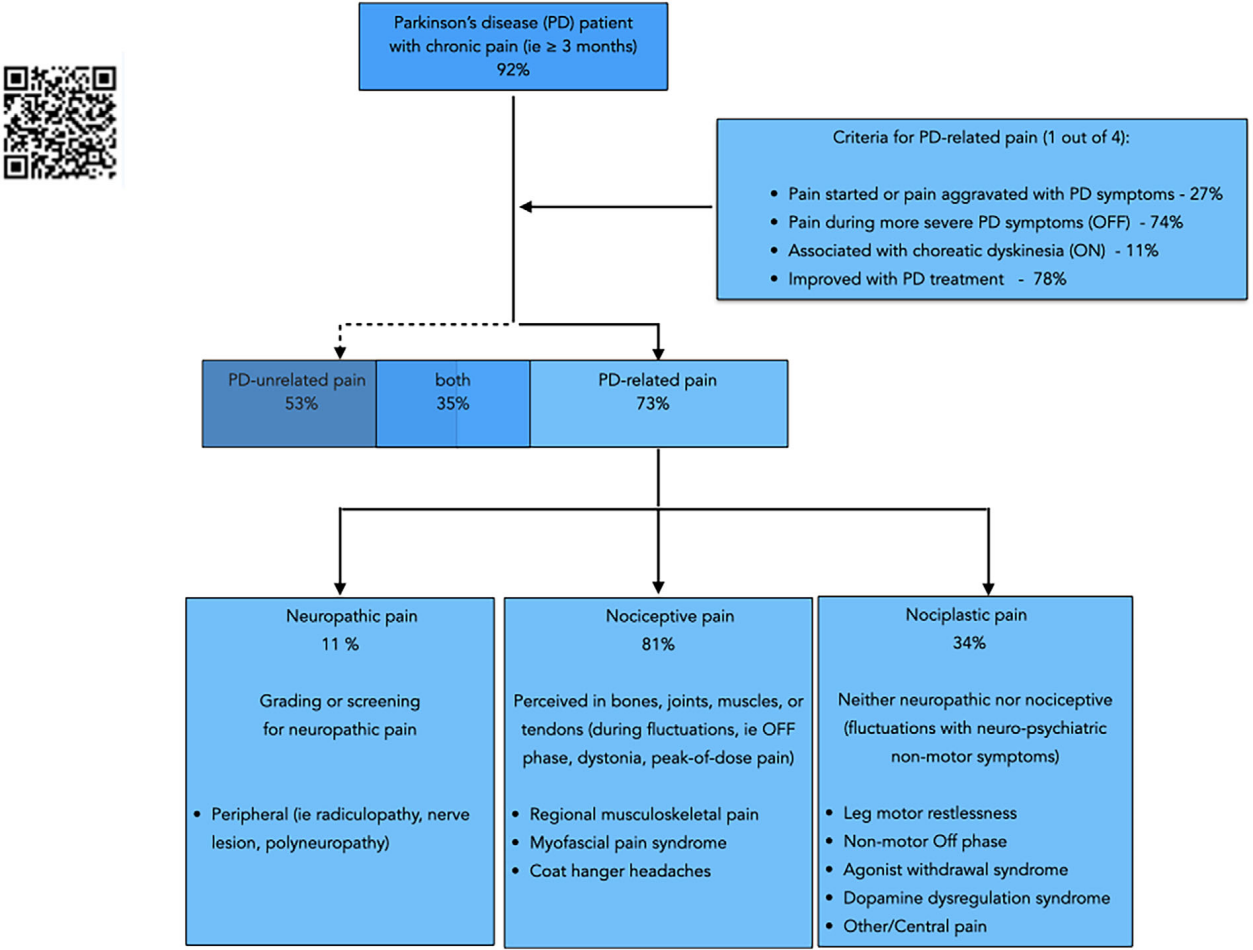
Algorithm of the Parkinson's Disease Pain Classification System (PD‐PCS) to distinguish PD‐related and PD‐unrelated pains before assigning the pain mechanism and the associated pain syndrome (modified with permission from Tinazzi et al.^2^), including the respective frequencies in 120 patients with PD. The QR code leads to an online application with a short version of the PD‐PCS questionnaire.^6^

Of the 120 patients, 110 (92%) presented with chronic pain, 88 (73%) with PD‐related pain, and 64 (53%) with PD‐unrelated pain (Fig. 1). Of those, 42 patients (35%) presented with both types of pain, 46 (38%) with only PD‐related pain, and 22 (18%) with only PD‐unrelated pain. The patients reported 221 pains (1.8 ± 1.0 per patient), with 74 (61.7%) patients reporting more than 1 pain. Of the 221 pains, 132 (60%) were PD related, whereas 89 (40%) pains were not associated with PD (Table 2). Table 3 presents the main characteristics of patients with PD‐related pain only, with PD‐unrelated pain only, with both types of pain, and without any chronic pain. Patients with PD‐related pain had higher MDS‐UPDRS IV scores, more frequent and severe dyskinesias, more *off* time, more fluctuations, and a higher mean PD‐PCS score. The scores of NMSs (ie, depression, anxiety, and cognition) do not differ statistically among the different groups. Quality of life was significantly reduced in patients with pain, without differences between chronic pain types. Tukey's HSD post hoc test could not identify significant differences between the groups, probably due to insufficient statistical power. There was a weak correlation between the PD‐PCS and PDQ‐8 scores (r = 0.25, *P* < 0.01) but no correlation between the PD‐PCS and HADS scores (anxiety: r = 0.12, *P* = 0.18; depression: r = 0.11, *P* = 0.20). Only 29 patients with pain (26.3%) were treated with analgesics and were similarly distributed between the groups.

**TABLE 2:** Characteristics of PD‐related and non‐PD‐related pains

	Non‐PD related	PD related	*P*‐value
	(N = 89)	(N = 132)	
Nociceptive	62 (89.9%)	91 (68.9%)	<0.001
Neuropathic	6 (8.7%)	10 (7.6%)	
Nociplastic	1 (1.4%)	31 (23.5%)	
Missing data	20	0	
All pains	(N = 69)	(N = 132)	
Positive DN4	7 (10.3%)	16 (12.1%)	0.881
0–10 cm VAS score	5.64 ± 1.78	5.59 ± 2.33	0.854
Frequency	2.15 ± 0.803	2.36 ± 0.755	0.078
Impact	2.15 ± 0.803	2.14 ± 0.814	0.929
PD‐PCS score	27.4 ± 18.9	31.6 ± 24.2	0.183
Nociceptive pain	(N = 62)	(N = 91)	
Positive DN4	5 (8.2%)	5 (5.5%)	0.745
0–10 cm VAS score	5.70 ± 1.83	6.08 ± 1.94	0.222
Frequency	2.13 ± 0.812	2.43 ± 0.717	0.023
Impact	2.15 ± 0.820	2.18 ± 0.787	0.836
PD‐PCS score	27.6 ± 19.4	36.2 ± 23.8	0.016
Neuropathic	(N = 6)	(N = 10)	
Positive DN4	2 (33.3%)	8 (80.0%)	0.182
0–10 cm VAS score	5.00 ± 1.26	6.20 ± 1.92	0.154
Frequency	2.17 ± 0.753	2.40 ± 0.699	0.551
Impact	2.00 ± 0.632	2.50 ± 0.707	0.169
PD‐PCS score	20.8 ± 9.04	39.2 ± 25.6	0.061
Nociplastic	(N = 1)	(N = 31)	
Positive DN4	0	3 (9.7%)	–
0–10 cm VAS score	6	3.94 ± 2.78	–
Frequency	3	2.13 ± 0.860	–
Impact	3	1.90 ± 0.885	–
PD‐PCS score	54	15.8 ± 17.5	–

Abbreviations: PD, Parkinson's disease; DN4, Questionnaire Douleur Neuropathique 4; VAS, Visual Analogue Scale; PD‐PCS, Parkinson's Disease Pain Classification System.

**TABLE 3:** Characteristics of PD patients with or without PD‐related or PD‐unrelated pains

Number of patients	No pain	Only non‐PD‐related pain	Only PD‐related pain	Both	*P*‐value
	(N = 10)	(N = 22)	(N = 46)	(N = 42)	
Type of pain					
Nociceptive	–	10 (45.5%)	37 (80.4%)	41 (97.6%)	<0.001
Neuropathic	–	0	7 (15.2%)	9 (21.4%)	0.058
Nociplastic	–	0	18 (39.1%)	12 (28.6%)	0.001
Mean PD‐PCS total score	–	7.21 ± 11.6	24.6 ± 19.8^a^	20.2 ± 14.1^a^	<0.001
Age	67.6 ± 10.8	72.1 ± 8.22	69.0 ± 9.22	70.3 ± 7.65	0.436
Sex					0.503
Male	5 (50.0%)	16 (72.7%)	26 (56.5%)	27 (64.3%)	
Female	5 (50.0%)	6 (27.3%)	20 (43.5%)	15 (35.7%)	
Years since diagnosis	6.02 ± 6.91	5.51 ± 3.20	7.93 ± 4.53	7.72 ± 5.36	0.199
LDED	656 ± 388	782 ± 368	885 ± 366	864 ± 483	0.379
Analgesics	2 (20.0%)	6 (27.3%)	10 (21.7%)	13 (31.0%)	0.758
MDS‐UPDRS III	24.1 ± 9.39	30.7 ± 14.8	30.2 ± 14.0	31.0 ± 13.6	0.544
MDS‐UPDRS IV	1.25 ± 1.75	1.27 ± 2.07	5.13 ± 3.641^b^	5.52 ± 4.58^b^	<0.001
Duration of dyskinesia	0.12 ± 0.35	0	0.52 ± 0.84^a^	0.43 ± 0.70	0.021
Disability due to dyskinesia	0.25 ± 0.46	0.32 ± 0.57	0.82 ± 0.53^a^, ^b^	0.81 ± 0.59^b^, ^c^	<0.001
*Off* time during waking day	0	0.09 ± 0.29	0.79 ± 1.1	0.93 ± 1.26^a^	0.006
MMSE	28.3 ± 1.70	28.6 ± 1.00	27.9 ± 2.08	28.7 ± 1.48	0.106
PDQ‐8	5.70 ± 3.16	7.27 ± 5.01	10.1 ± 5.95	9.02 ± 4.36	0.037
Hospital Anxiety Scale	5.20 ± 1.32	4.50 ± 4.27	6.20 ± 3.90	5.98 ± 4.22	0.379
Abnormal	1 (10.0%)	3 (13.6%)	15 (32.6%)	12 (28.6%)	0.235
Hospital Depression Scale	3.90 ± 3.54	5.59 ± 3.81	5.96 ± 3.23	5.45 ± 3.30	0.386
Abnormal	2 (20.0%)	5 (22.7%)	12 (26.1%)	11 (26.2%)	0.968
WOQ‐9 total	2.40 ± 2.01	2.36 ± 1.68	5.17 ± 1.94^a^, ^b^	4.79 ± 2.34^a^, ^b^, ^c^	<0.001
At least 1 wearing‐off criterion positive	9 (90.0%)	17 (77.3%)	45 (97.8%)	36 (92.3%)	0.04
McGill Pain Questionnaire					
Affective score	0	1.64 ± 2.26	3.61 ± 2.63^a^, ^b^	2.93 ± 2.31	0.002
Sensory score	0	7.23 ± 3.56^b^	10.9 ± 6.87^a^, ^b^	12.1 ± 5.84^a^, ^b^	<0.001
Total score	0	8.86 ± 4.72	14.5 ± 8.45^a^, ^b^	15.0 ± 7.49^a^, ^b^	<0.001

Abbreviations: PD, Parkinson's disease; PD‐PCS, Parkinson's Disease Pain Classification System; LDED, levodopa equivalent dose; MDS‐UPDRS, Movement Disorder Society‐unified Parkinson's Disease Rating Scale; MMSE, Mini‐Mental State Examination; PDQ‐8, Parkinson's Disease Questionnaire 8‐Item version; WOQ‐9, Wearing‐Off Questionnaire 9‐Item version.

^a^ Contrasts analysis: *P* < 0.05 versus only non‐PD‐related pain group.

^b^ Contrasts analysis: *P* < 0.05 versus no pain group.

^c^ Contrasts analysis: *P* < 0.05 versus only PD‐related pain group.

**FIG. 2:**
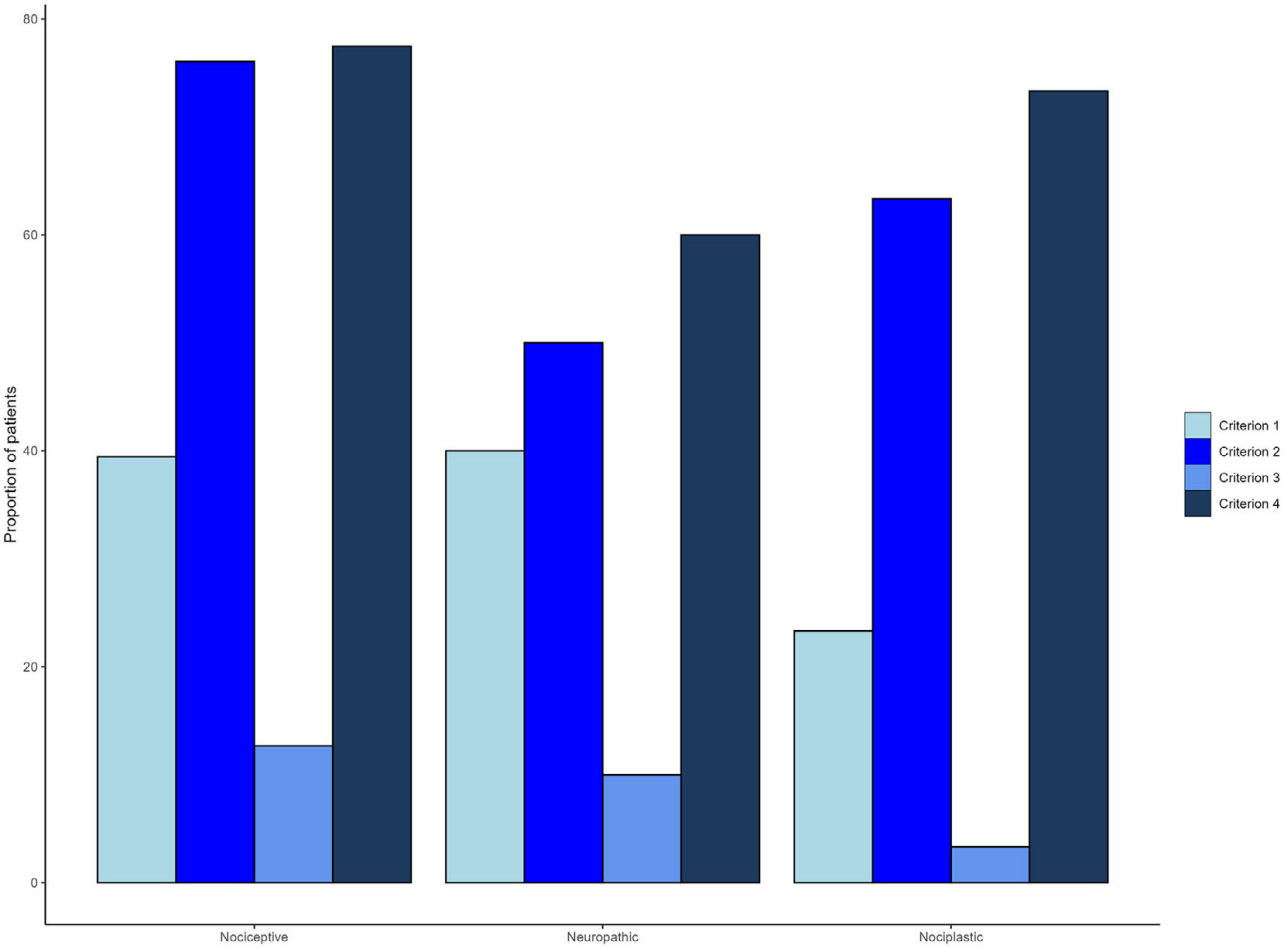
Frequency of the criteria defining PD (Parkinson's disease)–related pain for each pain category, according to the PD Pain Classification System (PD‐PCS). There were no significant differences in the frequency of each criterion per type of pain: criterion 1 (pain at disease onset or aggravated by PD), *P* = 0.379; criterion 2 (pain at the *off* phase), *P* = 0.332; criterion 3 (pain during dyskinesia), *P* = 0.243; criterion 4 (pain improvement with dopaminergic treatment) *P* = 0.602.

Nociceptive pains were the most common mechanistic type in patients with both PD‐related and PD‐unrelated pain, being significantly more frequent in the latter (68.9% vs. 89.9%, *P* < 0.001). Nociplastic pains were significantly more common in people with PD‐related pain (23.5% vs. 1.4%, *P* < 0.01). Regarding severity, PD‐related nociceptive pain was rated more severe than PD‐unrelated nociceptive pain (*P* = 0.016) (Table 2). The causes of nociceptive pain that were not related to PD were as follows: headaches (n = 7), cervical spine syndrome (n = 6), lower back pain (n = 19), joint pain (n = 24; upper extremities n = 13, lower extremities n = 11), peripheral arteriopathy (n = 3), calf cramps (n = 8), restless legs syndrome (n = 1), and myopathy (n = 1). All neuropathic pains were accounted for by polyneuropathy or neuropathy (n = 6), whereas fibromyalgia accounted for 1 nociplastic pain (n = 1). Higher chronic pain scores were given for nociceptive and neuropathic pains than for nociplastic pain (*P* < 0.001) (Figure 2). There were no other significant differences between groups (Table 4).

**TABLE 4:** Comparison between types of PD‐related pains

	Nociceptive	Neuropathic	Nociplastic	*P*‐value
	(N = 91)	(N = 10)	(N = 31)	
0–10 cm VAS score	6.08 ± 1.94	6.20 ± 1.92	3.94 ± 2.78	<0.001
Frequency	2.43 ± 0.717	2.40 ± 0.699	2.13 ± 0.860	0.176
Impact	2.18 ± 0.787	2.50 ± 0.707	1.90 ± 0.885	0.092
PD‐PCS score	36.2 ± 23.8	39.2 ± 25.6	15.8 ± 17.5	<0.001

Abbreviations: PD, Parkinson's disease; VAS, Visual Analogue Scale; PD‐PCS, Parkinson's Disease Pain Classification System.

### Associations between PD‐Related and PD‐Unrelated Pains

Correlation analysis showed a significant moderate correlation between the mean PD‐PCS severity score of PD‐related and PD‐unrelated pains (r = 0.42, *P* = 0.048) when both occurred in the same patients (35% of the participants). The McGill total score and its sensory and affective subscores were higher in PD‐related pain compared with unrelated pain, as were the total score and the sensory subscores when both pains co‐occurred (Table 3).

### Diagnostic Criteria for PD‐Related Pains

Of the 88 patients with PD‐related pain, independently of their mechanistic descriptor, “pain at motor symptom onset or aggravated by the disease” was reported by 24 patients (27.3%), “pain at the *off* phase” by 65 (73.9%), “pain during dyskinesia” by 10 (11.4%), and “pain responding to dopaminergic treatment” by 69 (78.4%). All criteria, except “pain during dyskinesia,” occurred with different prevalences together (Table 5). Pain improvement with dopaminergic treatment and pain at the worsening of motor symptoms occurred in 13.3%, 30.0%, and 31.0% of patients with nociceptive, neuropathic, and nociplastic pains, respectively (*P* = 0.267) (Table 5).

**TABLE 5:** PD patients meeting each PD‐PCS criterion for PD‐related pain by pain mechanism

	Nociceptive (N = 91)	Neuropathic (N = 10)	Nociplastic (N = 31)	*P*‐value
Criteria 1: pain at motor symptom onset or aggravated by the disease	32 (35.2%)	4 (40.0%)	7 (22.6%)	0.379
Criteria 2: pain at the *off* phase	64 (70.3%)	5 (50.0%)	19 (61.3%)	0.332
Criteria 3: pain during dyskinesia	13 (14.3%)	1 (10.0%)	1 (3.2%)	0.243
Criteria 4: pain responding to dopaminergic treatment	68 (74.7%)	6 (60.0%)	23 (74.2%)	0.602
*P*‐value*	<0.001	0.328	<0.001	
Combination of criteria				
1 and 2	6 (20.0%)	1 (10.0%)	18 (25.4%)	0.267
1 and 3	0	1 (10.0%)	4 (5.6%)	0.285
1 and 4	4 (13.3%)	3 (30.0%)	22 (31.0%)	0.267
2 and 3	0	0	0	–
2 and 4	14 (46.7%)	3 (30.0%)	43 (60.6%)	0.267
3 and 4	0	1 (10%)	0	–

Abbreviations: PD, Parkinson's disease; PD‐PCS, Parkinson's Disease Pain Classification System.

^*^ Chi‐sq test comparing the rate of proportions of the criteria per type of pain.

Nociceptive pain was reported by 71 (80.7%) patients, neuropathic pain by 10 (11.3%), and nociplastic pain by 30 (34.1%). Table 5 presents the number of patients fulfilling each PD‐PCS criteria for PD‐related pain, segregated by pain category. The prevalence of each category did not differ by criterion. However, nociceptive and nociplastic pains were highly prevalent in the *off* phases and responded well to dopaminergic treatment (*P* < 0.001 in both cases).

## Discussion

This study assessed the prevalence of the criteria used for defining chronic pain as being PD related or PD unrelated, and further compared the mutual occurrence and influence of both diagnoses. From 120 patients with PD, 92% presented with at least 1 chronic pain (1.8 ± 1.0); 73% had PD‐related pain, 53% had PD‐unrelated pain, and 35% presented with both types at the same time.

As previously shown, patients with PD‐related pain had more frequent and more severe dyskinesias and more off time.^6^ The NMSs assessed (anxiety, depression, and cognition) did not differ significantly between the groups, but quality of life was reduced when pain was present. Pain was rated higher when PD‐related pain was present, and there was a significant moderate correlation between the severity of PD‐related and non‐PD‐related pains. As awareness of dopaminergic schedule adjustment is not thought to be as high in the context of chronic pain, we suggest that the low pain treatment rate of a quarter reflects neglect of this NMS.^10^, ^36^

Analyses of the criteria defining PD‐related pain showed that improvement with dopaminergic medication and pain in the *off* phases were the most common factors defining this association with PD. These 2 factors, as well as pain at the onset or exacerbation of the disease, often occur simultaneously, whereas pain during dyskinesia occurs independently.

Nociceptive and nociplastic PD‐related pain occurred with a frequency of 80% and 34% of the sample. Both were more common in the off phases and were more likely to respond to dopaminergic treatment than neuropathic pains, which had a frequency of 11%. There were no significant differences in the frequency of diagnostic criteria for the 3 mechanisms. Higher pain ratings were given for nociceptive and neuropathic pain than for nociplastic pain. It has been proposed that patients with nociceptive/MSK PD‐related pains have different cortical excitability and somatosensory profiles compared to PD‐related pains of nociplastic and neuropathic mechanisms,^37^ and that these factors may influence response to therapy.^38^

The distribution of PD‐related and non‐PD‐related pain syndromes was 73% and 53%, respectively. The data differ from the validation study with 77% and 22%, respectively, due to differences between the Brazilian and the Swiss patients.^6^ The study by Lee et al.^5^ reported a similar prevalence of PD‐related and PD‐unrelated pains of about 63% and 64%, respectively, but used different criteria (no other clear cause including medical history, more intense on the more affected side, responding to dopaminergic treatment, and meeting a Ford criterion (MSK pain, radicular or neuropathic pain, dystonia‐related pain, akathisia, central pain)).^39^ They further classified “PD treatment‐related causes (0.8%)” and “indirectly related causes (eg, pressure ulcers, injuries from falls) (8%)” and “multiple causes (4%)” and did not differentiate acute from chronic pain. In contrast to their study, PD‐related pain was rated worse than PD‐unrelated pain in the present sample, presumably because frequency and impact on daily living were also considered for the score.^5^

Compared to larger studies such as those by Silverdale et al.^40^ and Gosh et al.,^4^ female sex, younger age at onset (presumably due to a noradrenergic nonmotor subtype in early‐onset PD),^41^, ^42^ disease duration, sleep disturbances, autonomic dysfunction, anxiety, and depression were either not assessed or not affected significantly in the present sample, whereas motor fluctuation such as off phases and dyskinesia were also seen to be associated. As shown in further studies,^43^, ^44^ nociceptive (MSK) pain is also a major contributor to chronic PD‐related pain in the present sample.

This is the first study that investigates the criteria determining the association of a respective pain syndrome with PD as employed using the PD‐PCS and also proposed for ICD11 (IASP Classification of Chronic Pain for the International Classification of Diseases) for chronic secondary MSK pain associated with PD.^6^, ^45^ They derived from pathophysiological studies showing an increased pain sensitivity in the *off* phase, which was at least partially reversible by dopaminergic treatment.^46^, ^47^, ^48^, ^49^ They include previous proposals by Quinn et al. and the NMS scale and were defined by an international consortium of movement disorder and pain specialists,^15^, ^50^ without including pain on the more affected side. For the definition of chronic secondary MSK pain associated with PD for ICD11, a threshold of at least 30% improvement in pain with dopaminergic treatment and the exclusion of further causes have been introduced.^45^ As the criterion “pain at the beginning of PD or pain aggravated by PD” consists of 2 criteria, they could also be mentioned independently as proposed recently.^2^

In the present sample, pain in the *off* phase and positive effects of dopaminergic treatment defined PD‐related pain in most cases. However, pain starting together with PD symptoms or the worsening of previous pain by PD may also define PD‐related pain. It is therefore important to consider all 3 criteria, as each criterion may define the association of pain with PD. In contrast, pain during the *on* phase (mostly during dyskinesia) is rare but may increase the prevalence of pain in PD, as suggested by some experimental studies and a recent imaging study.^51^, ^52^, ^53^ The frequent association with motor fluctuations suggests that PD treatment regimens are often not optimized for pain control and that pain is still often neglected in clinical practice.^2^, ^54^

Using these criteria, the frequency of PD‐related pain can be compared to previous studies that reported a range from 60% to 90%.^5^, ^6^, ^40^, ^55^ However, the number of patients with non‐PD‐related pain is higher compared to the general population (53 vs. 18 [10%–29%]) and similar to the study by Lee et al.^5^, ^8^ A large number of these patients additionally present with PD‐related pain, which may influence the prevalence of non‐PD‐related pain as mutual influences were shown previously.^11^, ^12^, ^13^ This is conceivable because, for example, shoulder stiffness or postural abnormalities can lead to shoulder or low‐back pathology, respectively. This interaction can also be inferred from the present data, as a moderate correlation was found between the severity of both types of pain.

Differences from the first validation study may also be explained by the fact that we also assessed up to 4 pains, but more than 1 pain in each category. Another limitation is the sample size and the fact that only 1 ethnic group was studied in Switzerland. Previous studies have shown that white people are more likely to be prescribed analgesics, particularly opioids. This suggests that there may be differences in pain perception, reporting, and prescription practices between ethnic groups.^56^ Although the sample size was sufficient to investigate some differences between PD‐related and non‐PD‐related pain, it was not large enough to assess all contributing and associated factors. The criterion of pain at disease onset or aggravated by the disease is likely to be subject to recall bias.

Chronic pain associated with PD is a common NMS that may be assessed using the PD‐PCS questionnaire. It includes comprehensive criteria for the association of chronic pain with PD, the 3 different pain categories, as well as an assessment of pain frequency and impact on daily living.^2^, ^6^, ^33^, ^57^, ^58^ Therefore, PD‐unrelated pain often occurs concurrently with PD‐related pain, and they exhibit reciprocal influences. *Off*‐phase pain, dopaminergic treatment effects, and pain associated with or exacerbated by PD are the most frequent criteria, suggesting relatively low dopaminergic stimulation as the main driver of chronic pain in PD. *On*‐phase pain (often associated with choreiform dyskinesia) tends to be isolated and rare. A movement diary may be helpful in adjusting dopaminergic treatment before pain medication is used (for treatment recommendations see references Tinazzi et al. and Mylius et al.).^2^, ^54^

## Author Roles

1. Research project: A. Conception, B. Organization, C. Execution;
2. Statistical analysis: A. Design, B. Execution, C. Review and critique;
3. Manuscript preparation: A. Writing of the first draft, B. Review and critique.

1: 1C, 2C, 3B

2: 1C, 2C, 3B

3: 1C, 2C, 3B

4: 1C, 2C, 3B

5: 1A, 1B, 1C, 2C, 3B

6: 1C, 2C, 3B

7: 2C, 3B

8: 1C, 2C, 3B

9: 2C, 3B

10: 1A, 1B, 2A, 2C, 3A, 3B

11: 1A, 2A, 2B, 2C, 3A, 3B

12: 1A, 1B, 1C, 2A, 2C, 3A, 3B

## Disclosures

**Ethical Compliance Statement**: The study protocol was approved by the local institutional review boards in St. Gallen and Lausanne (BASEC: 00502). Written informed consent was obtained from all participants. We confirm that we have read the journal's position on issues involved in ethical publication and affirm that this work is consistent with those guidelines.

**Funding Sources and Conflicts of Interest**: The study was supported by grants from Parkinson Schweiz, Zambon, and Mundipharma. The authors declared no conflicts of interest. Part of the data has been presented as poster at the EFIC Congress in Budapest 2023 and the MDS Congress 2024 in Philadelphia.

**Financial Disclosures for the Previous 12 Months**: N.H., F.B., G.K., J.C.M., D.H.B., R.G., M.T., D.C.A., and J.H. report no disclosures related to this work. J.F.B. received speaker honoraria from Bial, Spirig, AbbVie, Merz, and Medtronic. D.H.B. is funded by the Swiss National Science Fund and received speaker and consulting honoraria from Bial and Spirig. S.P.L. served as a consultant for Elea Laboratorios (Argentina), has stock options in TeleNeuro Solutions LLC (NJ, USA), and received grants from Agencia de Promoción Científica y Técnica (Argentina). V.M. has received a grant from Parkinson Switzerland. He received honoraria from AbbVie, Zambon, and Bial, and he served as a consultant for Bial.

## Data Availability

The data that support the findings of this study are available from the corresponding author upon reasonable request.
